# Exploring dropout in internet-delivered cognitive behavioral therapy for insomnia: A secondary analysis of prevalence, self-reported reasons, and baseline and intervention data as predictors

**DOI:** 10.1016/j.ijchp.2025.100598

**Published:** 2025-06-28

**Authors:** Laura Simon, Lisa Steinmetz, Eileen Bendig, Ann-Marie Küchler, Dieter Riemann, David Daniel Ebert, Kai Spiegelhalder, Harald Baumeister

**Affiliations:** a Institute of Psychology and Education, Department of Clinical Psychology and Psychotherapy, Ulm University, Ulm, Germany; b Department of Psychiatry and Psychotherapy, Medical Center – University of Freiburg, Faculty of Medicine, University of Freiburg, Germany; c GET.ON Institut für Online Gesundheitstrainings GmbH (operating under the registered brand ‘HelloBetter’), Hamburg, Germany; d Department of Sport and Health Sciences, Technical University of Munich, Munich, Germany

**Keywords:** Insomnia, Digital health, Dropout, Engagement, Completion

## Abstract

**Introduction:**

Internet-delivered cognitive behavioral therapy for insomnia (iCBT-I) is an effective treatment. However, dropout is a common challenge in digital therapeutics. This study examines dropout in iCBT-I by analyzing reported reasons for dropout and investigating whether baseline variables and intervention usage data can predict dropout.

**Methods:**

This is an exploratory secondary analysis of a clinical trial investigating a stepped care model for insomnia featuring an eight-module iCBT-I. Reasons for dropout from the iCBT-I were assessed via self-developed items in follow-up surveys, and a dropout survey was sent to all patients who had not completed at least seven modules of the iCBT-I within 12 weeks. The proportion of respondents who agreed with the respective items was calculated. Additionally, bivariate models were specified to explore whether baseline variables and intervention usage data can predict dropout.

**Results:**

The patients included in this sub-study had a mean age of 49.3 (SD=13.0), with 73.4 % identifying as female. At pre-treatment, their mean insomnia severity was 18.6 (SD=3.9). Among the 233 patients, 103 (44.2 %) were categorized as dropouts. The most frequently reported reasons for dropout were distractions from daily life, the perception of the content not being useful, and difficulties resuming after a break. None of the examined baseline variables significantly predicted dropout, whereas the time needed to complete the first module (OR=1.16; 95 %CI=1.08–1.27) and the number of sleep diary entries in the first week (OR=0.73; 95 %CI=0.65–0.80) significantly predicted dropout.

**Discussion:**

This study highlights dropout as a relevant challenge in iCBT-I, affecting over 40 % of patients. Self-reported reasons indicate the importance of compatibility with distractions from daily life and perceived effectiveness. The prediction models suggest that dropout risk profiles can be developed based on first-week treatment data. Future research should focus on validating such models to improve effectiveness and user retention.

## Introduction

Cognitive behavioral therapy for insomnia (CBT-I) is recommended as the first-line treatment for insomnia disorder (Qaseem et al., 2016; Riemann et al., 2017, 2023). Providing CBT-I in conventional face-to-face settings remains challenging due to the limited number of trained CBT-I providers (Koffel, Bramoweth & Ulmer 2018). Given the strong evidence supporting internet-delivered CBT-I (iCBT-I) (Hasan et al. 2022; Simon et al. 2023; van Straten et al. 2018), the recent update to the European Insomnia Guidelines (Riemann et al. 2023) has included iCBT-I as an A-level recommended treatment. However, one challenge commonly discussed in the treatment setting of digital therapeutics is dropout.

Two decades ago, Eysenbach (2005) introduced the "Law of Attrition", which describes the observation that many individuals prematurely discontinue eHealth trials or cease using the intervention. When discussing dropout, it is essential to recognize that dropout comprises several constructs. It can be distinguished between study dropout (i.e., failure to complete surveys for the scientific evaluation of a trial) and intervention dropout (i.e., failure to complete the intervention itself) (Eysenbach 2005). However, within the construct of intervention dropout, there is no consensus on its definition. For instance, some studies define it as failing to complete all available modules of an intervention, while others establish a minimum number of modules that need to be completed. Furthermore, dropout and retention are closely linked to the construct of adherence, which, in the context of CBT-I, can be defined as following treatment recommendations (Mellor et al. 2022; Steinmetz et al. 2023). As adherence requires active engagement with the digital therapeutic beyond merely reading the content, it is essential to clearly distinguish between these concepts. In the remainder of the manuscript, "dropout" refers to intervention dropout.

A systematic review synthesizing research on retention in digital therapeutics identified three domains influencing retention: (1) patient characteristics (e.g., demographics, mental health status, obligations in daily life), (2) program characteristics (e.g., type of content, perceived fit, perceived usefulness), and (3) technology and environmental factors (e.g., usability of technology, privacy, and confidentiality) (Borghouts et al. 2021). In terms of mental health status, this systematic review found that while the severity of mental health symptoms increased patients' interest in digital therapeutics, mental health symptoms such as low mood and fatigue can act as barriers to retention. This finding is particularly relevant given that daytime impairments, such as fatigue, low mood, irritability, and impairments in cognitive performance, are symptoms of insomnia (Riemann et al. 2023; Wardle-Pinkston, Slavish & Taylor 2019). Hence, these impairments may make it challenging for individuals with insomnia to complete iCBT-I, as it is a self-help treatment that relies heavily on the patient's initiative and sustained attention. Due to the substantial heterogeneity in definitions and rates of dropout, quantifying dropout in iCBT-I studies remains challenging. Reported dropout rates range from as low as 5 % (Pillai et al. 2015) to over 50 % (Nam et al. 2023). A systematic review of five studies investigating iCBT-I that defined dropout as failing to complete the entire intervention found an average dropout rate of 36 % (Horsch et al. 2015). While premature discontinuation is a broader issue in psychotherapy, affecting approximately one in five patients in conventional face-to-face therapy (Swift & Greenberg 2012), in CBT-I, the risks for dropout appear to be particularly high in group therapy and iCBT-I (Simon et al. 2023). Moreover, it should be considered that dropout in routine conditions may be even higher than in controlled trials, given the lack of monitoring and attention by a study team in these contexts (Baumel, Edan & Kane 2019; Fleming et al. 2018; Knowles et al. 2015).

While it may not be essential for patients to complete all modules of an iCBT-I, sufficient exposure to the therapeutic content seems crucial. This is supported by findings from a meta-regression examining the relationship between intervention completion/adherence and treatment outcomes, showing that intervention completion/adherence can increase the effect size by 0.2 (Horsch et al. 2015). Moreover, it is important to consider that dropout may not only impede improvement due to insufficient exposure to essential therapeutic content but could also negatively impact patients' future motivation to seek help if needed.

Hence, dropout is an important aspect of iCBT-I that warrants more attention to improve the treatment of insomnia. Several aspects are worth further investigation. One key area is identifying participant characteristics predictive of dropout, which could optimize treatment allocation. Among the baseline variables examined, several studies have found that less severe sleep problems may be associated with dropout. Specifically, higher sleep efficiency (Ström, Pettersson & Andersson 2004), greater total sleep time (Hebert et al. 2010; Ström et al. 2004; Yeung et al. 2015), shorter wake after sleep onset (WASO) (Nam et al. 2023; Ström et al. 2004), lower insomnia severity (Nam et al. 2023), and lower expectations about sleep (Nam et al. 2023) have been associated with dropout in iCBT-I. Moreover, there is some evidence that comorbid mental health conditions (Hebert et al. 2010) and the presence of depressive symptoms (Yeung et al. 2015) are associated with dropout. However, given the variability in study results and the use of differing statistical methods to identify predictors, the generalizability of these predictors remains uncertain. Therefore, further investigations of whether these or other baseline variables predict dropout are needed.

Another potentially complementary approach to identifying baseline predictors could involve the investigation of the predictive utility of intervention usage metrics that are available early in the intervention. Digital therapeutics offer the advantage of collecting large volumes of data, often without requiring additional patient input. For instance, the time a patient needs to complete a module is frequently recorded in the intervention platforms. Some evidence exists that both in insomnia (Bremer et al. 2020) and in other mental health areas, as in depression (Moshe et al. 2022), the time needed to complete the first module is associated with dropout. Given that this information is available early in the intervention, this would allow for early adaptations to better address patients' needs and possibly mitigate dropout.

One strategy commonly discussed to mitigate dropout is providing human therapeutic guidance. In the broader field of digital therapeutics, qualitative research suggests that such guidance can reduce dropout; however, results in quantitative studies are inconsistent (Boucher & Raiker 2024). A network meta-analysis comparing guided (i.e., some form of human guidance) and unguided (i.e., no human guidance/only technical guidance) iCBT-I has found a comparable risk of dropout for these two treatment settings (Simon et al. 2023). However, data from clinical trials may not be fully generalizable to routine care, particularly as it can be assumed that even in unguided iCBT-I, the involvement of a study team introduces an implicit form of human accountability. These involvements are also likely to vary between trials. Therefore, to control for such confounding factors, it is essential to conduct head-to-head comparisons to understand how varying intensities of therapeutic support influence dropout. In addition to guided and unguided approaches, a resource-efficient alternative called guidance-on-demand has been proposed to provide support in digital therapeutics, where guidance is only provided when explicitly requested by the patients (Berger et al. 2011).

This secondary data analysis aims to investigate the following research questions to further the understanding of dropout in iCBT-I, which was defined as completing <80 % of the iCBT-I program within 12 weeks.

1. What are the overall intervention completion rates across three intervention groups of an iCBT-I that vary in their therapeutic guidance?
2. What are the reported reasons for dropout, and does the descriptive data highlight distinct explanatory patterns?
3. Can baseline variables predict dropout?
4. Can data collected during the first module (e.g., intervention usage data, sleep diary data) be used to predict dropout?

Given the exploratory nature of this study, no a priori hypotheses have been set. However, the analysis was informed by general working assumptions based on prior literature. Specifically, we assumed that intervention completion rates would be higher in groups receiving more intense therapeutic guidance. Regarding baseline predictors of dropout, we expected that individuals with a lower insomnia severity would be more likely to discontinue the iCBT-I. Additionally, for data collected during the first module, we anticipated that indicators such as sleep diary entries showing less severe insomnia symptoms, as well as a longer time taken to complete the first module, would be predictive of dropout.

## Methods

### Study design & setting

This sub-study is an exploratory secondary analysis of the cluster-randomized trial GET Sleep, investigating a stepped care model for insomnia (Spiegelhalder et al. 2022). The definition of intervention dropout as completing <80 % within 12 weeks was pre-defined in the study protocol of the main study (Spiegelhalder et al. 2022). However, the specific data analysis strategy was not pre-specified. The stepped care model consisted of three steps, and patients could progress to the next step if they perceived that the current step was not sufficient to treat their symptoms. The first step of the stepped care model consisted of diagnostics and psychoeducation delivered by a general care physician. In the second step, the patients received access to an iCBT-I. In the third step, patients received specialized face-to-face treatment (e.g., psychotherapeutic, sleep medicine, or psychiatric care).

The iCBT-I in the second step was structured into eight modules, each intended to last approximately 45 to 60 min. The modules encompassed the following treatment components which were based on CBT-I manuals: psychoeducation and goal setting (module 1), sleep restriction therapy (module 2), help with maintaining sleep restriction therapy (module 3), stimulus control therapy (module 4), cognitive therapy (module 5), strategies to handle rumination and worry (module 6), relaxation techniques (module 7). The last module included reflection and relapse prevention. Patients were suggested to complete one module per week, resulting in a total intervention duration of eight weeks. However, patients had the flexibility to progress through the program at their own pace.

GET Sleep investigated three intervention groups that differed in the intensity of therapeutic guidance provided by an e-coach (i.e., trained psychologist under supervision) during the iCBT-I and one control group (i.e., treatment as usual). All patients in the intervention groups received an introductory call before commencing the iCBT-I and a final call upon completing the iCBT-I. In the guidance condition, patients received written, semi-standardized feedback from their e-coach after each module. The guidance-on-demand condition allowed patients to initiate contact with their e-coach as needed, while the e-coach did not proactively reach out. E-coaches were instructed to spend 10 to 20 min drafting a semi-standardized response. The focus was on ensuring that the feedback was formulated in a validating manner, emphasizing (partial) successes. In the basic condition, communication between patients and the e-coach was limited to the introductory and final call. Furthermore, the iCBT-I automatically sent up to three reminders to prompt the initiation or completion of the next module in all intervention groups.

### Participants

Patients were recruited in Germany through advertisements (e.g., online, print, broadcast) and postal mailings sent by a collaborating insurance provider. To be included in the cluster-randomized trial of GET Sleep, patients had to be 18 years or older and have a diagnosis of non-organic insomnia (ICD-10 F51.0) or insomnia (ICD-10 G47.0) according to the ICD-10 (World Health Organization 1992), which their general care physician assessed. Please refer to Spiegelhalder et al. (2022) for detailed information on the inclusion and exclusion criteria. The main study included 333 patients. As GET Sleep followed a stepped care approach, where not all patients needed to progress to the second step, this secondary analysis included only participants from the intervention groups who had initiated the iCBT-I. For the cluster-randomized controlled trial, general care physicians were the unit of randomization. The combination of this level of randomization and recruitment challenges led to an unequal distribution of patients across the intervention groups.

### Variables

For the main GET Sleep study, self-report data were assessed online via LimeSurvey (LimeSurvey GmBH n.d.) at baseline, four weeks after baseline, twelve weeks after baseline, and six months after baseline.

#### Baseline variables

The following data from the baseline survey were used in this sub-study: Insomnia severity was assessed using the Insomnia Severity Index (ISI; Bastien, Vallières & Morin 2001). The total score of the ISI can range from 0 to 28, and the ISI has demonstrated good internal consistency (Cronbach's alpha = 0.70 to 0.90; Bastien et al. 2001; Gagnon et al. 2013; Morin et al. 2011). Depressive symptoms were assessed using the Quick Inventory of Depressive Symptomatology - Self Report (QIDS-SR16; Rush et al. 2003). The total score for the QIDS-SR16 ranges from 0 to 27, and the internal consistency is good (Cronbach's alpha = 0.86; Trujols et al. 2018). Anxiety symptoms were assessed using the Generalized Anxiety Disorder-7 questionnaire (GAD-7; Spitzer et al. 2006). The total score for the GAD-7 ranges from 0 to 21, and the GAD-7 has demonstrated good internal consistency (Cronbach's alpha = 0.89; Löwe et al. 2008). Somatic symptoms were assessed using the Somatic Symptom Scale-8 (SSS-8; Gierk et al. 2014). The total score of the SSS-8 ranges from 0 to 32, and the internal consistency is good (Cronbach's alpha = 0.81; Gierk et al. 2014). Dysfunctional beliefs and attitudes about sleep were assessed via the ten-item version of the Dysfunctional Beliefs and Attitudes about Sleep Scale (DBAS-10; Espie et al. 2000). The total score of the DBAS-10 ranges from 0 to 10, and the internal consistency is moderate (Cronbach's alpha = 0.69; Espie et al. 2000). Pre-Sleep Arousal was assessed via the Pre-Sleep Arousal Scale (PSAS; Nicassio et al. 1985). The PSAS somatic and cognitive subscales can take scores from 8 to 40 and show good to excellent internal consistencies (Cronbach's alpha = 0.80, 0.94, respectively; Gieselmann, De Jong-Meyer & Pietrowsky 2012). Symptoms of stress were assessed using the Perceived Stress Scale (PSS; Cohen, Kamarck & Mermelstein 1983). The total score of the PSS can range from 0 to 50, and the internal consistency has been shown to be good (Cronbach's alpha = 0.84; Klein et al. 2016).

Moreover, self-report data on age, gender, and relationship status (in a relationship vs. single vs. divorced/widowed) and education status from the baseline survey were used in this sub-study. The education status was assessed by asking participants about their highest educational degree. These self-report answers were categorized in line with the Comparative Analyses of Social Mobility in Industrial Nations (CASMIN) classification into three categories (i.e., low, moderate, and high; Schneider 2016).

#### Reasons for dropout from survey data

Patients who did not complete at least 80 % of the iCBT-I within 12 weeks of starting it (i.e., fewer than seven modules, as recorded on the iCBT-I platform) were classified as dropouts. These patients were invited via email to a specific dropout survey. As the survey's first question, patients were asked if they still intended to complete the iCBT-I. Following, the patients were shown a self-developed 35-item Likert-scale (1 to 5, from "does not apply at all" to "does completely apply") questionnaire to identify reasons for intervention dropout. These items were formulated based on the Health Action Process Approach (Schwarzer, Lippke & Luszczynska 2011). Please refer to Supplemental Material 1 for an overview of the assessed items. One item explored whether other reasons, not previously listed, hindered the completion of the iCBT-I. If applicable, patients were presented with a follow-up question listing potential reasons, such as vacation, illness, hospital stay, or other factors.

Additionally, at each follow-up survey (i.e., four weeks, twelve weeks, and six months after the baseline survey), conducted as part of the general evaluation of GET Sleep, patients were asked whether they had prematurely discontinued the iCBT-I. If they answered yes, they were presented with a multiple-choice question to identify reasons for discontinuing the iCBT-I. The reasons comprised 18 predefined options (e.g., lack of time, dissatisfaction with e-coaching) along with an additional "other reasons" category. Supplementary Material 3 displays all assessed items.

The multiple-choice questions in both the follow-up and dropout surveys addressed similar themes related to the reasons for dropout. However, the dropout survey had a higher level of detail, also capturing facilitating factors and utilizing a different question format with a Likert scale. Data from both sources were included in the study to maximize the response rate from this difficult-to-access population.

#### Intervention data

Additionally, both passive (i.e., not requiring additional input from the patients) and active (i.e., requiring input from the patients) intervention data were collected. Each module's start and end times were passively collected through the iCBT-I platform. After completing the first module, patients were asked when they planned to work on the next module. Moreover, sleep diary data from the online sleep diary were collected for this sub-study. Sleep diary data of the first week for the time of going to bed, falling asleep, waking up, getting up, and any wake times after sleep onset were considered.

### Statistical methods and data handling

Dropout was operationalized as a binary outcome (dropout vs. completer) based on passively collected module start and end times from the iCBT-I platform, with those completing <80 % of the iCBT-I program within 12 weeks classified as dropouts. This definition was based on the specific content of the iCBT-I program, with the final module focusing solely on reflection and relapse prevention. The time criterion was set at 12 weeks to reflect the recommended pacing of one module per week, while also accommodating the flexibility patients had to proceed at their own pace, including potential interruptions due to vacation or illness. The proportions of completers and dropouts in the intervention groups were compared using Fisher's exact test due to unequal cell distribution between the intervention groups.

Reasons for dropout were analyzed separately using responses from dropout and follow-up surveys. The first question of the dropout survey, in which patients were asked whether they intended to complete the iCBT-I, was used to identify those who expressed a continued intention to complete the iCBT-I. Intervention usage data were matched to these patients to analyze whether these patients ultimately completed the iCBT-I. The responses from the self-developed questionnaire in the dropout survey were dichotomized as follows: values of 1 and 2 were categorized as 'disagree', while values of 4 and 5 were categorized as 'agree'. Responses with a value of 3 were considered neutral. The proportion of respondents who agreed with the respective items was calculated. As patients could report premature discontinuation of the iCBT-I at any follow-up survey, the earliest instance at which a patient indicated discontinuation and answered the multiple-choice question on reasons for dropout was used for the respective patient for the analysis. The relevance of the reported reasons for dropout was determined based on whether they were among the most frequently reported and appeared in both surveys. However, due to the preliminary nature of the study, no specific threshold was predefined.

For the prediction models, baseline variables and intervention data were processed as follows: The baseline variables' scores were calculated according to the instructions provided for the respective instrument. For the PSAS, the sum scores for the cognitive domain (PSAS_COGN) and the somatic domain (PSAS_SOMA) were calculated. From the intervention data, the time needed to complete the first module was calculated for each patient. Moreover, the time difference between the date patients completed the first module and their intended date to work on the second module was calculated. From the online sleep diary, the number of sleep diary entries per patient for the first week was calculated. Following the sleep diary data underwent a plausibility check to identify any implausible values. Implausible values (i.e., negative total sleep time, total sleep time over 900 min, time in bed over 960 min) were removed. The weekly averages for the time in bed (TIB; i.e., duration between bedtime and time the person got up), TST (i.e., duration between falling asleep and waking up subtracted by WASO), sleep efficiency (SE; total sleep time divided by time in bed multiplied by 100), and WASO were calculated.

Bivariate analyses were conducted to assess the odds ratios (ORs) for predicting the binary outcome of dropout using the following baseline variables: age, gender, relationship status, education status, ISI, GAD-7, QIDS-SR16, DBAS, SSS-8, PSAS_SOMA, PSAS_COGN, and PSS. Moreover, bivariate analyses to predict dropout were conducted using the following intervention data: time needed to complete the first module, time difference between the completion of the first module and intended start date for the second module, number of sleep diary entries in the first week, TST, TIB, SE, and WASO. Bivariate analyses were chosen to identify potential predictors for use in more complex models in future research. For the bivariate analyses, the assumption of linearity of the logit was evaluated visually for all continuous predictor variables. The visual inspections indicated no violations of this assumption for any predictor variable; therefore, no transformations were deemed necessary. Statistical significance for the examined predictors was determined based on whether the 95 % confidence interval (CI) of the OR excluded 1.0. Given the exploratory nature of the study and the assumption that even small effects may carry clinical relevance, particularly within cumulative risk models, significant ORs relatively close to 1.0 were also considered potentially meaningful. However, replication in future studies is necessary to fully establish these findings' clinical relevance.

As this was an exploratory study, no adjustments for multiple testing were made. All patients were required to complete the baseline survey for inclusion, resulting in no missing baseline data. All available data were utilized for all other data points, such as the dropout survey and intervention data.

### Ethics

The study protocol received approval from the Ethics Committee of the Medical Center—University of Freiburg as well as from the Ethics Committee of the State Chamber of Physicians ('Landesärztekammer Baden-Württemberg'). Furthermore, the data protection concept for this study was approved by the data protection officers of both the Medical Center—University of Freiburg and Ulm University. Informed consent was obtained from all patients before inclusion in the study. Patients received a payment of €15 for completing online surveys at four and twelve weeks post-baseline and €20 for completing the online survey six months post-baseline. No payment was provided for completing the baseline survey or the dropout survey.

## Results

### Sample description

A total of 233 patients had at least initiated the iCBT-I and were included in this sub-study, with 172 (73.8 %) identifying as female. The patients had a moderate insomnia severity (mean = 18.6; SD = 3.9). A total of 148 patients were in the guidance condition, 53 in the guidance-on-demand condition, and 32 in the basic condition. Detailed characteristics of the patients at baseline are provided in Table 1.

**Table 1:** Baseline variables.

	n ( %) or mean (SD)
Age	49.3 (13.0)
Female ( %)	172 (73.82 %)
Relationship	
In a relationship	189 (81.2 %)
Single	29 (12.5 %)
Divorced or widowed	15 (6.4 %)
Education (CASMN)^1^	
Low	6 (2.6 %)
Moderate	97 (41.6 %)
High	130 (55.8 %)
Insomnia Symptoms (ISI)^2^	18.6 (3.9)
Depressive Symptoms (QIDS-SR16)^3^	8.4 (3.9)
Anxiety Symptoms (GAD-7)^4^	6.7 (3.9)
Somatic symptoms (SSS-8)^5^	10.9 (4.3)
Dysfunctional beliefs about sleep (DBAS-10)^6^	5.9 (1.5)
Pre Sleep Arousal Scale – somatic subscale (PSAS_SOMA)^7^	11.6 (3.8)
Pre Sleep Arousal Scale – cognitive subscale (PSAS_COGN)^7^	17.0 (7.2)
Perceived Stress Scale (PSS)^8^	28.2 (6.6)

*Note*.

1 : Education was categorized in line with the Comparative Analyses of Social Mobility in Industrial Nations into low, moderate, and high (Schneider 2016).

2 : Insomnia Severity Index (Bastien et al. 2001).

3 : Quick Inventory of Depressive Symptomatology - Self Report (Rush et al. 2003).

4 : Generalized Anxiety Disorder 7 questionnaire (Spitzer et al. 2006).

5 : Somatic Symptom Scale-8 (Gierk et al. 2014).

6 : Dysfunctional Beliefs and Attitudes about Sleep Scale-10 (Espie et al. 2000).

7 : Pre-Sleep Arousal Scale (Nicassio et al. 1985).

8 : Perceived Stress Scale (Cohen et al. 1983).

### iCBT-I completion

**Fig. 1:**
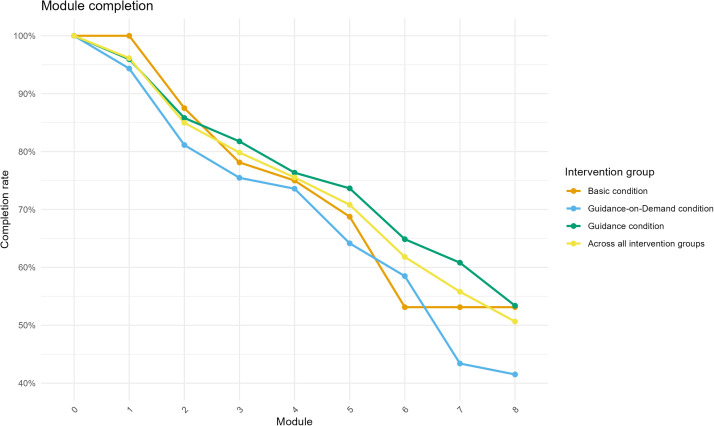
Module completion rates across intervention groups. *Note.* Only modules completed within 12 weeks were considered.

One hundred thirty patients (55.8 %) were classified as completers (i.e., completing at least seven modules within 12 weeks of initiating the iCBT-I). Conversely, 103 (44.2 %) patients were categorized as dropouts. Considering the intervention groups, 90 patients (60.8 %) in the guidance condition, 23 patients (43.4 %) in the guidance-on-demand condition, and 17 patients (53.1 %) in the basic condition were categorized as completers. The Fisher's Exact Test comparing the ratio of iCBT-I completion between the intervention groups was not significant (*p* = 0.09). When the 12-week time criterion was not applied, 144 patients (61.8 %) completed all eight modules. Fig. 1 displays the percentage of patients who have completed the respective module within 12 weeks.

### Reported reasons for dropout

All 103 patients categorized as non-completers were invited to complete the dropout survey. Of these, 38 (36.9 %) responded to the dropout survey; complete survey data were available from 33 patients. Follow-up survey data from 37 patients who prematurely discontinued the intervention and provided reasons for dropout were available. Eleven patients provided answers in both the follow-up survey and the dropout survey.

High levels of agreement were observed on items assessing intention and motivation, with endorsement from over 90 % of patients. In the dropout survey, distractions from daily life, difficulties resuming treatment after a break, and the perception that iCBT-I was not helpful in improving sleep problems were the most frequently reported reasons. The ten most frequently endorsed dropout reasons are depicted in Table 2. Importantly, 66.7 % of patients indicated other reasons for discontinuation. For the follow-up item on other reasons, eight patients reported vacation, eleven illness, two a hospital stay, and nine their job as reasons for discontinuation. In the dropout survey, 25 patients indicated they still intended to complete the iCBT-I. Of these patients, one completed seven modules, and nine completed all eight. In the follow-up surveys, the most frequently reported reasons were occupational duties (40.5 %), the perception of the content not being useful (32.4 %), and lack of motivation (29.7 %).

**Table 2:** Most frequently endorsed items from the dropout survey.

	% (fully) agree
Too many distractions from daily life to complete the next module	65.4
Difficulties resuming iCBT-I after a long break	46.4
iCBT-I not helpful for improving sleep-related symptoms	43.5
Lack of time to regularly participate in iCBT-I	41.7
iCBT-I too impersonal	39.3
iCBT-I not helpful for improving well-being	35.7
iCBT-I insufficient for reducing distress	33.3
Technical difficulties	28.6
Lack of motivation to complete iCBT-I	26.9
Dissatisfaction E-Coaching	25.0

*Note.* iCBT-*I* = internet-delivered cognitive behavioral therapy for insomnia (in the survey labeled as online training). Only items considered to contribute to dropout are listed here, whereas facilitating factors for retention, which were also assessed, were not considered. The responses from the self-developed questionnaire in the dropout survey were dichotomized as follows: values of 1 and 2 were categorized as 'disagree', while values of 4 and 5 were categorized as '(fully) agree'. Responses with a value of 3 were considered neutral.

Supplementary Material 1 displays the percentage of respondents who (fully) agreed (i.e., rating of 4 and 5) with the respective item of the dropout survey. Analyses of the open-ended follow-up questions can be found in Supplementary Material 2. An overview of the prevalence of the assessed reasons for dropout in the follow-up surveys is presented in Supplementary Material 3.

### Predicting dropout with baseline variables and intervention data

Table 3 presents the bivariate models specified to predict dropout based on participants' baseline characteristics. In the bivariate analyses, none of the examined baseline variables emerged as significant predictors of dropout.

**Table 3:** Predictors of dropout from baseline variables.

**Predictors**	**Bivariate model** **OR (95 %CI)**	***p* Value**
Age	0.99 (0.97; 1.01)	0.15
Gender (male)	1.31 (0.73; 2.36)	0.36
Relationship Single vs. in a relationship Divorced/widowed vs. in a Relationship	0.55 (0.23; 1.24) 1.84 (0.64; 5.67)	0.16 0.27
Education (CASMIN)^1^ High vs. low/moderate	0.73 (0.43; 1.22)	0.23
Insomnia symptoms (ISI)^2^	1.01 (0.95; 1.08)	0.68
Depressive symptoms (QIDS-SR16)^3^	1.00 (0.93; 1.06)	0.86
Anxiety symptoms (GAD-7)^4^	1.01 (0.95; 1.08)	0.72
Somatic symptoms (SSS-8)^5^	0.99 (0.93; 1.05)	0.66
Dysfunctional beliefs about sleep (DBAS-10)^6^	1.09 (0.92; 1.30)	0.33
Pre Sleep Arousal Scale – somatic subscale (PSAS_SOMA)^7^	1.01 (0.94; 1.08)	0.82
Pre Sleep Arousal Scale – cognitive subscale (PSAS_COGN)^7^	1.02 (0.98; 1.06)	0.32
Perceived Stress (PSS)^8^	1.00 (0.96; 1.04)	0.89

*Note.* These analyses were based on data from all 233 patients who had initiated the iCBT-I. OR = Odds ratio. CI = Confidence interval.

1 : Education was categorized in line with the Comparative Analyses of Social Mobility in Industrial Nations into low, moderate, and high (Schneider 2016).

2 : Insomnia Severity Index (Bastien et al. 2001).

3 : Quick Inventory of Depressive Symptomatology - Self Report (Rush et al. 2003).

4 : Generalized Anxiety Disorder 7 questionnaire (Spitzer et al. 2006).

5 : Somatic Symptom Scale-8 (Gierk et al. 2014).

6 : Dysfunctional Beliefs and Attitudes about Sleep Scale-10 (Espie et al. 2000).

7 : Pre-Sleep Arousal Scale (Nicassio et al. 1985).

8 : Perceived Stress Scale (Cohen et al. 1983).

Table 4 presents the bivariate models predicting dropout based on intervention data. In the bivariate analyses, the time needed to complete the first module (OR = 1.16; 95 % CI = 1.08–1.27) and the number of sleep diary entries in the first week (OR = 0.73; 95 % CI = 0.65–0.80) emerged as significant predictors, whereas none of the calculated sleep metrics from the sleep diary data were significant.

**Table 4:** Predictors of dropout from intervention data.

**Predictors**	**Bivariate model** **OR (95 %CI)**	***p* Value**
Time needed to complete the first module	1.16 (1.08; 1.27)*	*p* < 0.001
Number of sleep diary entries in the first week	0.73 (0.65; 0.80)*	*p* < 0.001
Time difference between the completion of the first module and the intended start of the second module	0.96 (0.85; 1.03)	0.34
Total sleep time	1.00 (1.00; 1.00)	0.68
Time in bed	1.00 (1.00; 1.01)	0.82
Sleep efficiency	0.99 (0.97; 1.01)	0.54
Wake after sleep onset	1.00 (0.99; 1.01)	0.56

*Note.* Analyses were based on the following sample sizes: 226 for the time needed to complete the first module, 233 for the number of sleep diary entries in the first week, 116 for the time difference between completing the first module and the intended start of the second module, and 177 for total sleep time, time in bed, sleep efficiency, and wake after sleep onset. OR = Odds ratio. CI = Confidence interval. Plausible entries from the first week of the iCBT-I in the online sleep diary were used to calculate the means for total sleep time, time in bed, sleep efficiency, and wake after sleep onset.

## Discussion

This study aimed to investigate dropout in an iCBT-I, which had three intervention groups that differed in the intensity of the therapeutic guidance. Overall, 55.8 % of the patients were categorized as completers, defined as completing at least seven of the eight modules within 12 weeks of initiating the iCBT-I. Descriptively, the intervention group receiving the most intensive therapeutic guidance (guidance condition) achieved the highest completion rate (60.8 %). However, Fisher's Exact Test indicated no statistically significant differences between the intervention groups. The most prominent self-reported reasons for dropout included too many distractions from daily life, difficulties resuming after a break, and the perception of the iCBT-I as not being useful. None of the examined baseline variables were predictive of dropout. Instead, the time needed to complete the first module and the number of sleep diary entries submitted during the first week emerged as significant predictors of dropout. However, further replications are needed to confirm the findings and clarify their clinical relevance

### What are the overall intervention completion rates across three intervention groups of an iCBT-I that vary in their therapeutic guidance?

In the existing literature, reported dropout rates are very heterogeneous. With a dropout rate of 44.2 %, the rate of this trial falls on the higher end of reported dropout rates. However, it is important to note that researchers have used varying criteria to define dropout, such as completing all modules, completing a specific number of modules, or meeting these thresholds within a specified time frame. This inconsistency makes direct comparisons of non-usage dropout rates across studies challenging. Nonetheless, the finding that over 40 % of patients were categorized as dropouts across the intervention groups highlights the urgent need to understand and address dropout in iCBT-I.

In the broader literature on digital therapeutics, quantitative research on whether human support mitigates dropout has been mixed (Boucher & Raiker 2024), and for iCBT-I meta-analytic evidence yielded similar risks of dropout in guided and unguided iCBT-I (Simon et al. 2023). This study provides a meaningful contribution to understanding how therapeutic guidance affects dropout, as it investigates the same intervention across three intervention groups that only differed in the level of therapeutic guidance. Hence, contextual factors like the support provided by a study team or how the iCBT-I was structured were held constant. Although the observation of this study requires confirmation in adequately powered studies, the trend indicating that the guided condition had the lowest dropout rate supports the notion that human support may help reduce dropout. Nevertheless, as this study was still conducted in a clinical trial, factors related to the trial context may have influenced dropout, particularly in the basic condition. In this context, it is important to note that even patients in the basic group had an introductory call with an e-coach. Therefore, this group was not entirely unguided. It seems plausible that this initial contact may have already had an effect, and without it, the differences between the basic and guided conditions would likely have been more pronounced. This underscores the need to investigate dropout data from iCBT-I implementations in real-world conditions and in settings where no guidance is provided.

Interestingly, the guidance-on-demand condition, intended to mitigate dropout while being more resource-efficient, descriptively yielded the highest dropout rate. A potential explanation, derived from a qualitative sub-study of GET Sleep (Simon et al. 2025) might be that patients in the guidance-on-demand condition did not actively reach out to their e-coach despite experiencing the additional need for support. Hence, further research is required to identify how guidance-on-demand might need to be altered to become a resource-efficient, retention-promoting strategy. This could, for example, feature pro-active outreaches by e-coaches if patients have been disengaged for an extended period or outreaches from the e-coaches during particularly challenging modules, such as during the introduction of sleep restriction therapy.

### What are the reported reasons for dropout, and does the descriptive data highlight distinct explanatory patterns?

#### Distraction from daily life, occupational duties, and lack of time

Distractions from daily life, occupational duties, and lack of time were among the most commonly reported reasons for dropout. This is in line with literature that has identified time constraints as an important barrier to engagement with digital therapeutics (Boucher & Raiker 2024). The flexibility of iCBT-I (i.e., regarding the time and place patients work on the modules) may convey the misleading impression that the treatment is not time-intensive, leading patients to believe it can be easily integrated into their daily routine without significant effort or need for prioritization. However, the core treatment components of iCBT-I often require patients to make substantial adjustments to their daily lives and habits (Agnew et al. 2020). Hence, it appears important to ensure that patients undergoing iCBT-I are prepared to prioritize iCBT-I during the intervention period over potential demands from daily life and their occupation.

#### Difficulties resuming iCBT-I after a break

Difficulties in resuming after a break were identified as the second most common reason for dropout in the dropout survey. Moreover, follow-up items revealed that illness and vacations were frequently cited as reasons for discontinuation. These findings suggest that breaks of any kind may present important barriers to the retention of iCBT-I. To mitigate this, patients may benefit from additional support, such as proactive outreach from the program or e-coach, along with intensified guidance, to help them resume iCBT-I after an interruption.

#### Perceived effectiveness

Moreover, items indicating that patients perceived iCBT-I as ineffective in improving both sleep-related symptoms and overall well-being were frequently cited. In the context of iCBT-I, the frequent occurrence of short-term negative effects (Kyle et al. 2011) might further contribute to this impression. Indeed, literature has consistently identified perceived effectiveness as an crucial facilitator for retaining digital therapeutics (Borghouts et al. 2021; Boucher & Raiker 2024). Hence, it might be important to assess the perceived effectiveness of iCBT-I as part of ongoing process monitoring. This would also be interesting, as little is known about how perceived effectiveness relates to outcomes typically assessed as primary or secondary endpoints in research, such as insomnia severity and sleep efficiency.

#### Insufficient personal contact/ personal fit with treatment setting

Interestingly, insufficient personal contact was reported more frequently than dissatisfaction with e-coaching across both the follow-up and dropout surveys. This discrepancy suggests that perceptions of insufficient personal contact are not necessarily linked to dissatisfaction with the e-coaching itself. While further investigation is needed to clarify what constitutes "insufficient personal contact", it may reflect a broader misalignment between the treatment setting and individual preferences. This idea is further supported by follow-up responses in which some participants expressed reluctance to spend additional time in front of a screen.

#### Sufficient improvements

While dropout is often viewed negatively, it is also possible that some patients discontinue treatment after achieving sufficient improvement, suggesting that completing the entire iCBT-I program may not represent the optimal therapeutic dosage for these individuals (Boucher & Raiker 2024). The findings of our study indicate that although it may not be prevalent, this phenomenon does occur. According to self-report data, 8–10 % of participants discontinued treatment due to sufficient improvements. Hence, some individuals may have benefited from the iCBT-I by the time of their discontinuation, and viewing their dropout as negative would be a misconception (Boucher & Raiker 2024). However, further research is needed to assess whether the improvements observed in quick responders are sustained over the long term.

### Can baseline variables predict dropout?

In contrast to other studies on predicting dropout in iCBT-I (Hebert et al. 2010; Nam et al. 2023; Ström et al. 2004; Yeung et al. 2015), which identified WASO, TST, depressive symptoms, dysfunctional beliefs about sleep, and a higher insomnia severity as potential predictors of intervention dropout, none of the investigated baseline variables were predictive of dropout in this study. A possible explanation for this divergence is that our study defined dropout more leniently than others, requiring only 80 % of module completion (i.e., seven modules), whereas other studies required the completion of all modules. However, this definition was chosen because it might better reflect real-world usage and because patients were introduced to all components of CBT-I at this point. While our findings require further research in adequately powered studies, it may be more promising to investigate other baseline variables (e.g., demands from daily life and effort individuals are willing to allocate to iCBT-I) or to rely on data that is available early during the intervention to identify individuals at risk for dropout.

### Can data collected during the first module (e.g., intervention usage data, sleep diary data) be used to predict dropout?

In the bivariate models, a longer time to complete the first module (OR = 1.16) and fewer sleep diary entries during the first week (OR = 0.73) emerged as significant predictors of dropout. In the context of the bivariate models, this indicates that for each additional day required to complete the first module, the odds of dropout increase by 16 %, while each additional completed sleep diary entry is associated with a 27 % decrease in the odds of dropout. The finding that a longer time needed to complete the first module predicts dropout is consistent with previous studies (Bremer et al. 2020; Moshe et al. 2022). However, given the exploratory nature of this study, these findings should be interpreted with caution. Further replications are needed to confirm the results and clarify their clinical relevance. Similar to baseline variables, sleep indices calculated from the sleep diary were not predictive of dropout.

Additionally, the time difference between the first module's completion and the second module's intended start did not predict dropout. This finding aligns with the high agreement on items addressing motivation and intention in the dropout survey. Moreover, while 25 patients in the dropout survey stated they intended to complete the iCBT-I, only nine ultimately completed the iCBT-I. Together, these observations highlight the potential limitations of self-reports on motivation and intention in accurately identifying individuals at risk of dropout. This aligns with a previous study that did not find an association between dropout and intention in iCBT-I (Hebert et al. 2010). A possible explanation could be that such statements are confounded by biases such as social desirability and thus do not truly reflect the intention of these individuals. In contrast, passively assessed variables, such as the number of sleep diary entries completed in the first week and the time required to complete the first module, may be less conflated by such biases.

Importantly, these passively assessed variables are collected early in the intervention and require minimal processing (e.g., no extensive validation of entered sleep diary values before calculating indices). As such, these variables could serve as indicators for identifying participants at risk of dropout after the first week of treatment. Given the modest effects of intervention usage data, future research should aim to identify additional variables that can help detect individuals at risk of dropout. In this context, it could be valuable to consider other demographic factors (e.g., e-health literacy, technical affinity, workload), clinical characteristics such as daytime symptoms of insomnia (e.g., fatigue, concentration difficulties), and to explore the predictive value of commonly reported reasons for dropout. Moreover, future research should focus on validating such risk prediction models. While the surveys on reasons for dropout examined intervention-related factors to some extent, it may also be important to explore in greater depth how factors such as persuasive design, model complexity, usability, and personalization contribute to dropout. In this context, considering that cognitive impairments are a common symptom of insomnia (Wardle-Pinkston et al. 2019) and that self-help interventions, such as iCBT-I, can place substantial cognitive demands on patients (Johansson et al. 2015), future research should investigate the extent to which cognitive impairments contribute to treatment dropout. Understanding this relationship could inform the design of future iCBT-I with a stronger emphasis on reducing cognitive load, building on preliminary research from other mental health domains that has demonstrated positive effects on retention (Sheeber et al. 2017).

Based on these findings, future iCBT-I could potentially be more tailored to individual needs; for example, by adaptively personalizing content (e.g., skipping or intensifying treatment components) and guidance (e.g., increasing support from an e-coach or incorporating onsite therapy) based on individual risk for intervention dropout (Forsell et al. 2019; Knowles et al. 2015).

### Limitations

This study provides valuable insights into dropout by examining it from multiple perspectives. However, several limitations must be acknowledged. First, dropout was operationalized as completing fewer than seven modules within 12 weeks. While this definition was made considering the content of the specific iCBT-I, this definition was not evidence-based. This underscores the need for further research to establish what constitutes a minimally effective therapeutic dosage of iCBT-I and how this might vary between varying combinations of treatment components. Second, there is a potential selection bias in the survey data. While the participation rate of 37 % in the dropout survey is relatively high, the remaining 63 % of eligible patients did not participate in the dropout survey. These 63 % represent individuals who fully disengaged from both the scientific evaluation and the iCBT-I. Thus, their perspectives would be particularly valuable, and more initiatives must be taken to follow these individuals in future studies. Furthermore, the prediction models did not include variables reflecting the reasons commonly reported for dropout. Therefore, future research should investigate whether these reasons, along with other demographic and clinical variables not included in our models, can serve as predictors of dropout. A further limitation is the imbalance in participant allocation among the intervention groups, which may have influenced the conclusions drawn from the group comparisons. Moreover, the predominantly female sample may limit the generalizability of these findings to other populations. For the follow-up surveys, data from the earliest time a patient indicated discontinuation of iCBT-I was used. Thus, the measurement time of this data varied, which could have led to distortions. Moreover, it has to be considered that the iCBT-I was embedded in a stepped-care model, which could have also led to confoundments. Finally, it is important to emphasize that this study was exploratory in nature and was not powered to test the specified prediction models. These limitations highlight the need for future studies specifically designed to investigate hypotheses related to dropout.

While research on dropout is important, it is crucial to recognize that completing a module does not necessarily reflect adherence to the recommended treatment content or meaningful engagement with it (Boucher & Raiker 2024). Thus, while the completion (of a certain number of) modules appears to be necessary, it may not be a sufficient condition for treatment success. Future research should focus on refining the operationalization of engagement. Nevertheless, investigating dropout provides an important initial step in advancing the understanding of challenges in iCBT-I.

## Conclusion

The results of this study underscore that dropout is a relevant challenge in iCBT-I, likely driven by diverse underlying causes. Importantly, this sub-study focused on dropout within the context of a clinical trial. To fully understand the scope and nuances of dropout, it is essential to examine real-world data and implement additional initiatives to gather data from patients who disengage entirely from iCBT-I. While baseline variables did not emerge as significant predictors of dropout, it might be promising to investigate whether the demands of daily life can predict dropout. Furthermore, a promising direction for future research is to explore first-week intervention usage metrics as a foundation for developing risk models for dropout. However, it appears essential to confirm these findings and to identify other variables predictive of dropout.

## Declaration of competing interest

DE has served as a consultant to/on the scientific advisory boards of Sanofi, Novartis, Minddistrict, Lantern, Schoen Kliniken, Ideamed and German health insurance companies (BARMER, Techniker Krankenkasse) and a number of federal chambers for psychotherapy. He is also shareholder of "GET.ON Institut für Online Gesundheitstrainings GmbH für Gesundheitstrainings online GmbH" (HelloBetter), which aims to implement scientific findings related to digital health interventions into routine care and is the provider of the intervention under investigation in this study. DR has received honoraria for speaking engagements from GET.ON Institut für Online Gesundheitstrainings GmbH, Heel and Idorsia. DR has received consultancy fees from GAIA.AG, Idorsia and X-trodes. HB reports to have received consultancy fees, fees for lectures or workshops from chambers of psychotherapists and training institutes for psychotherapists and license fees for an Internet intervention. All other authors have no conflicts of interest to declare.

## Appendix Supplementary materials

Image, application 1
