# Supplementary material for: Exploring dropout in internet-delivered cognitive behavioral therapy for insomnia: A secondary analysis of prevalence, self-reported reasons, and baseline and intervention data as predictors
Source: Int J Clin Health Psychol. 2025 Jun 28;25(3):100598. doi: 10.1016/j.ijchp.2025.100598 (PMC12269844; doi:10.1016/j.ijchp.2025.100598)
Supplement: Supplementary file 1 [file mmc1.docx]

Exploring reasons and predictors for dropout in an internet-delivered cognitive behavioral therapy for insomnia

Content

[Supplemental Material 1: Dropout survey 2](#_Toc189660314)

[Supplemental Material 2: Analyses follow-up questions dropout survey 3](#_Toc189660315)

[Supplemental Material 3: Reported reasons for dropout from follow-up survey 4](#_Toc189660316)

## Supplemental Material 1: Dropout survey

| **Supplementary Table 1.** Dropout survey | |
| --- | --- |
| **Item** | **% (fully) agree** |
| **Facilitating factors** |  |
| Motivation to participate in online training | 90.6 |
| Sufficient support from the study team to start online training | 90.3 |
| Sufficient information to start online training | 86.7 |
| Capability to regularly complete online training | 80.8 |
| Planned when and where do online training | 30.4 |
| Intention to complete online training regularly | 96.8 |
| Ideal environment to complete online training | 72.0 |
| Set date to work on the next module | 60.0 |
| Planned when to work on the next module | 56.0 |
| Online training met personal needs | 73.1 |
| **Hindering factors** |  |
| Content not useful | 25.0 |
| Online training not helpful for improving sleep-related symptoms | 43.5 |
| Online training not helpful for improving well-being | 35.7 |
| Online training insufficient for reducing distress | 33.3 |
| Dissatisfaction E-Coaching | 25.0 |
| Technical difficulties | 28.6 |
| Online training too complicated | 20.7 |
| Online training not enjoyable | 20.0 |
| Online training too impersonal | 39.3 |
| Through discontinuation, more time for other things | 15.6 |
| Too many distractions from daily life to complete the next module | 65.4 |
| Lack of time to regularly participate in online training | 41.7 |
| Neglected important things in life because of time spent on online training | 23.1 |
| Lack of motivation to complete online training | 26.9 |
| Online training had a negative impact | 12.1 |
| Online training too disturbing | 12.9 |
| Occurrence of negative effects | 10.0 |
| Advice from others to discontinue | 3.1 |
| Difficulties resuming online training after a long break | 46.4 |
| Doubts about the capability to complete online training after a break | 20.7 |
| Initiated other treatment (because of insomnia) | 3.2 |
| Initiated other treatment (other psychological issues) | 0.0 |
| Significant improvements due to other circumstances | 9.7 |
| Significant improvements due to online training | 8.7 |
| Other reasons | 66.7 |
| *Note.* iCBT-I = internet-delivered cognitive behavioral therapy for insomnia (in the survey labeled as online training). The responses from the self-developed questionnaire in the dropout survey were dichotomized as follows: values of 1 and 2 were categorized as 'disagree', while values of 4 and 5 were categorized as '(fully) agree'. Responses with a value of 3 were considered neutral. | |

## Supplemental Material 2: Analyses follow-up questions dropout survey

In the multiple-choice selection for "other reasons," eight patients noted vacation, eleven cited illness, two reported a hospital stay, and nine attributed discontinuation to their job. Patients who indicated additional reasons mentioned the following: five mentioned life circumstances (e.g., having a baby, changing jobs, hospitalization of a family member), two reported psychological problems, one stated that their sleep problems were not psychological (i.e., problems breathing, orthopedic issues and neuropathy), and one noted difficulty with the smartphone application. Another patient reported that while they found SRT potentially helpful, other components were not effective, but that they felt SRT would only be feasible during vacation. Additionally, this person expressed reluctance to spend time online after working on a computer all day.

## Supplemental Material 3: Reported reasons for dropout from follow-up survey

| **Supplementary Table 2.** Reported reasons for dropout from follow-up survey |  |
| --- | --- |
| **Reason** | **%** |
| Occupational duties | 40.5 |
| Content not useful | 32.4 |
| Lack of motivation | 29.7 |
| Insufficient personal contact | 27.0 |
| Technical difficulties | 27.0 |
| Other reasons | 24.3 |
| Lack of time | 21.6 |
| Symptoms too pronounced for iCBT-I | 18.9 |
| iCBT-I too strenuous | 13.5 |
| No time because of vacation | 13.5 |
| Illness | 10.8 |
| Dissatisfaction with e-coaching | 8.1 |
| Having found a more suitable treatment option | 8.1 |
| Feeling better (no need for iCBT-I) | 8.1 |
| Initiated psychotherapy | 8.1 |
| Hospitalization | 5.4 |
| Initiated medical treatment for psychological issues | 5.4 |
| Decided with the e-coach that the iCBT-I did not meet the needs | 5.4 |
| Occurrence of negative effects | 0.0 |
| *Note.* Reasons for dropout were analyzed using responses from the follow-up surveys. As patients could report the premature discontinuation of the iCBT-I each follow-up survey, the earliest time a patient indicated it and responded to the multiple-choice question on reasons for dropout was used for the analyses. As these reasons were shown in a multiple choice format, patients might have indicated more than one reason for non-completion. iCBT-I = internet-delivered cognitive behavioral therapy for insomnia (in the items in the survey labeled as online training). | |

Nine patients indicated in the follow-up surveys that "other reasons" were responsible for discontinuing the intervention. The responses to the subsequent open-ended question were categorized into the following themes: Four patients reported a limited personal fit with iCBT-I, two patients mentioned difficulties resuming after a break, and two patients cited waiting for psychotherapy. Other reasons included lack of time (n=1), comorbid symptoms (n=1), excessive screen time (n=1), perceived improvement (n=1), and life circumstances (n=1).
